# Molecular and cellular evidence for biased mitotic gene conversion in hybrid scallop

**DOI:** 10.1186/1471-2148-10-6

**Published:** 2010-01-11

**Authors:** Shi Wang, Lingling Zhang, Jingjie Hu, Zhenmin Bao, Zhanjiang Liu

**Affiliations:** 1 Key Laboratory of Marine Genetics and Breeding of Ministry of Education, Ocean University of China, Qingdao 266003, China; 2 The Fish Molecular Genetics and Biotechnology Laboratory, Aquatic Genomics Unit, Department of Fisheries and Allied Aquacultures and Program of Cell and Molecular Biosciences, Auburn University, Auburn, AL 36849, USA

## Abstract

**Background:**

Concerted evolution has been believed to account for homogenization of genes within multigene families. However, the exact mechanisms involved in the homogenization have been under debate. Use of interspecific hybrid system allows detection of greater level of sequence variation, and therefore, provide advantage for tracing the sequence changes. In this work, we have used an interspecific hybrid system of scallop to study the sequence homogenization processes of rRNA genes.

**Results:**

Through the use of a hybrid scallop system (*Chlamys farreri *♀ × *Argopecten irradians *♂), here we provide solid molecular and cellular evidence for homogenization of the rDNA sequences into maternal genotypes. The ITS regions of the rDNA of the two scallop species exhibit distinct sequences and thereby restriction fragment length polymorphism (RFLP) patterns, and such a difference was exploited to follow the parental ITS contributions in the F1 hybrid during early development using PCR-RFLP. The representation of the paternal ITS decreased gradually in the hybrid during the development of the hybrid, and almost diminished at the 14th day after fertilization while the representation of the maternal ITS gradually increased. Chromosomal-specific fluorescence *in situ *hybridization (FISH) analysis in the hybrid revealed the presence of maternal ITS sequences on the paternal ITS-bearing chromosomes, but not vice versa. Sequence analysis of the ITS region in the hybrid not only confirmed the maternally biased conversion, but also allowed the detection of six recombinant variants in the hybrid involving short recombination regions, suggesting that site-specific recombination may be involved in the maternally biased gene conversion.

**Conclusion:**

Taken together, these molecular and cellular evidences support rapid concerted gene evolution via maternally biased gene conversion. As such a process would lead to the expression of only one parental genotype, and have the opportunities to generate recombinant intermediates; this work may also have implications in novel hybrid zone alleles and genetic imprinting, as well as in concerted gene evolution. In the course of evolution, many species may have evolved involving some levels of hybridization, intra- or interspecific, the sex-biased sequence homogenization could have led to a greater role of one sex than the other in some species.

## Background

Concerted evolution is the tendency of the different genes in a gene family or cluster to evolve in concert, resulting in sequence homogenization among the members of the family [1]. Two primary mechanisms, unequal crossover and gene conversion, were believed to function for the homogenization of multigene families [2]. Extensive studies of the tandemly repeated ribosomal RNA (rRNA) genes suggest that unequal crossover is the major driving force in the evolution of the rRNA genes with sister chromatid exchange occurring more often than exchange between homologs. Gene conversion is also believed to play a role; however, direct evidence for its involvement has not been obtained [3]. However, these mechanisms can act to achieve apparently opposite results: they can correct and eliminate new variants and they can also promote the spread of new gene variants throughout individual gene clusters, among homologous and nonhomologous chromosomes, and within an interbreeding population [4]. In recent years, gene conversion has become more popular in many theoretical and experimental studies [5-8]. Although gene conversion in yeast can be explained by a DNA breakage followed by invasive DNA replication [9], the molecular mechanism of gene conversion in multigene families is not well understood particularly when sequence identity is patchy [1].

Within eukaryotic genomes, rDNA gene family exists in arrays (nucleolar organizer regions or NORs on one or more chromosomes) of varying length, ranging from a single gene in the case of *Tetrahymena *to several thousand copies per genome in some cereals [10]. Different subunits in rDNA family are known to evolve at varying rates, depending upon the degree of selective constraint operative on each. In these subunits, the internal transcribed spacer (ITS) is not internally repetitive and its evolving rate is between those of genes and non-transcribed region [11]. Therefore, ITS is an ideal region for the study of the homogenization processes of rDNA family [12].

Natural or synthetic allopolyploids and hybrids with clear and very recent ancestry are good models for studying concerted evolution of multigene families [6,13]. In order to study the homogenization process of rDNA family, we exploited heterogeneity of ITS sequences (over 20% sequence divergence) in a hybrid scallop (*Chlamys farreri *♀ × *Argopecten irradians *♂). The great sequence divergence would allow the homogenization process, if any, to be readily detected in the hybrid. Here we report the homogenization of ITS sequences in the hybrid by gene conversion, leading to the generation of novel hybrid alleles. The gene conversion is massively rapid during early development of the hybrid, and is biased toward the maternal copy. We provide direct evidence for gene conversion in the ITS region of the rDNA gene clusters that may have implications not only for concerted evolution, but also for the origin of hybrid zone novel alleles, and for genetic imprinting.

## Results

**Figure 1:**
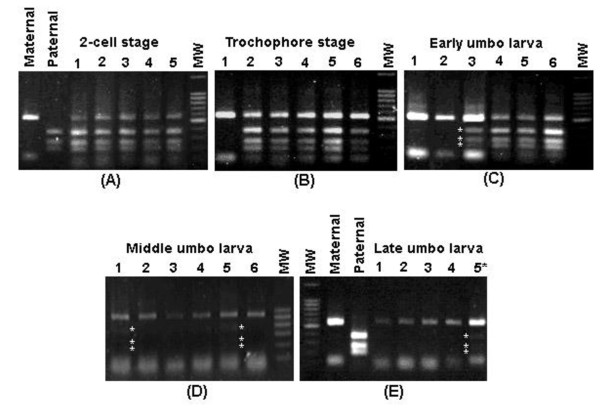
**PCR-RFLP analysis of make-up of the ITS region in the hybrid with restriction enzyme *Hae *III at early development**. Panel A exhibits PCR-RFLP analysis at the 2-cell stage (about 1 hour after fertilization) with samples from maternal parent *C. farreri *(Maternal), paternal parent *A. irradians *(Paternal), and samples from five random selected hybrid individuals (1 through 5), and molecular weight standard (MW). Panel B exhibits PCR-RFLP analysis at the trochophore stage (about 20 hour after fertilization) with samples from six random selected hybrid samples (1 through 6), and molecular weight standard (MW). Panel C exhibits PCR-RFLP analysis at the early umbo larva stage (about 4 days after fertilization) with samples from six random selected hybrid samples (1 through 6), and molecular weight standard (MW). Panel D exhibits PCR-RFLP analysis at the middle umbo larva stage (about 10 days after fertilization) with samples from six random selected hybrid individuals (1 through 6), and molecular weight standard (MW). Panel E exhibits PCR-RFLP analysis at the late umbo larva stage (about 14 days after fertilization) with samples from maternal parent (Maternal), paternal parent (Paternal), and samples from four random selected hybrid individuals (1 through 4) and a sample from mixed samples of multiple individuals (5*), and molecular weight standard (MW). Molecular weight standards were 100 bp DNA ladder.

PCR-RFLP analysis of ITS sequences provides a rapid diagnostic tool for species identification of *C. farreri *and *A. Irradians *[14]. In the hybrid generated by crossing *C. farreri *female with *A. irradians *male, the restriction patterns using restriction enzyme *Hae *III differentiate the maternal ITS from paternal ITS. The *C. farreri *maternal ITS should produce three PCR-RFLP fragments of 526 bp, 117 bp and 98 bp (741 bp in total), while the paternal *A. irradians *ITS should produce three PCR-RFLP fragments of 343 bp, 244 bp and 182 bp (769 bp in total). In order to assess dynamic variation of ITS constitution in the hybrid, PCR-RFLP was conducted at various early developmental stages. As shown in Figure 1, the proportion of the maternal and paternal alleles varied greatly with development. At the 2-cell stage, both the maternal and the paternal alleles were equally present (Figure 1A); at the trochophore stage (approximately 20 hours after fertilization), most larvae harbored alleles from both parents, while some (less than 5%) harbored only the maternal ITS (Figure 1B); at the early stage of umbo larvae (approximately 4 days after fertilization), most larvae still harbored alleles from both parents, but signal intensities of the restriction fragments representing the paternal *A. irradians *became significantly lower in some larvae, and the proportion of the larvae that possessed only the maternal ITS allele was increased (Figure 1C); at the middle stage of umbo larvae (approximately 10 days after fertilization), most larvae possessed only the maternal ITS allele, and even in those that still possessed the parental ITS allele (less than 30%), signal intensities of the restriction fragments representing the paternal allele of *A. irradians *were significantly lower (Figure 1D). At the late stage of umbo larvae (approximately 14 days after fertilization), the vast majority of larvae harbor only the maternal ITS allele as paternal allele was not evident when samples were analyzed individually (lanes 1 through 4, Figure 1E), although the restriction fragments from the paternal *A. irradians *allele could still be detected when multiple larvae were simultaneously included in PCR-RFLP analysis (lane 5, Figure 1E). In general, the proportion of the paternal ITS allele decreased gradually in the hybrid during the development. At the 14th day after fertilization, the paternal allele representation became extremely low (Figure 1).

**Figure 2:**
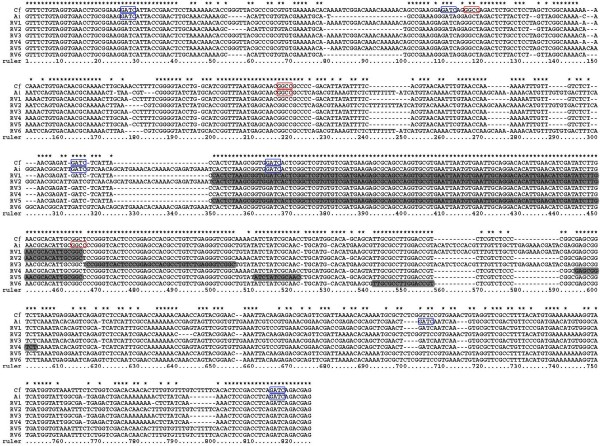
**Alignment of parental and 6 recombinant variants' sequences**. Cf: *C. farreri*; Ai: *A. irradians*; RV1-6: recombinant variants 1-6. Potential recombinant regions in recombinant variants are shown in black background. Asterisks show identical bases; dashes indicate alignment gaps. (ITS1: 34-350 bp; 5.8S: 351-506 bp; ITS2: 507-805 bp). Restriction sites for *Mbo *I GATC are indicated by a blue box while restriction sites for *Hae *III GGCC are indicated by a red box.

Recombinant Variants and Potential Recombinant Regions: When PCR product of multiple larvae at the trochophore stage was cloned, 6 recombinant variants were identified in about 200 randomly selected colonies with PCR-RFLP technique (restriction enzymes *Hae *III and *Mbo *I). The frequencies of the six variants are 6% (RV1), 3% (RV2), 1.5% (RV3), 1.5% (RV4), 1% (RV5) and 2% (RV6). These recombinant variants are composed of segmental sequences of *C. farreri *and *A. irradians*. These recombinant variants were probably intermediates of the paternal ITS undergoing biased gene conversion. Of the six recombinant variants, RV1, RV3, and RV4 had their first part of the amplified ITS region containing sequences from the maternal parent, and their second part of the amplified ITS region containing sequences from the paternal parent. In contrast, RV2 and RV6 contained their first part of the amplified ITS region containing sequences from the paternal parent, and the second part of the amplified ITS region containing sequences from the maternal parent. For RV5, the two terminal parts of the amplified ITS region containing sequences from the maternal parent, whereas the middle segment of approximately 50 bp containing sequences from the paternal allele (Figure 2).

**Figure 3:**
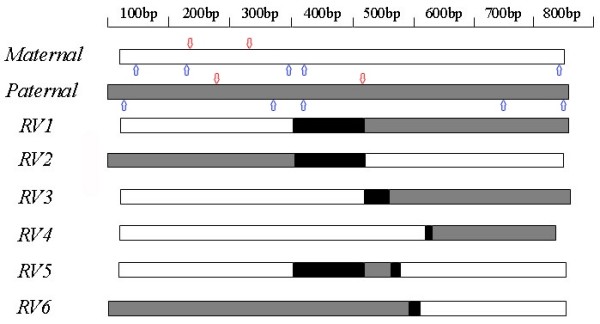
**Schematic presentation of the recombinant variants of the interspecific hybrid showing the origins of the sequences in the ITS region**. Open bar, sequences from maternal parent; sketched bar, sequences from paternal parent. Restriction sites for *Mbo *I are indicated by blue arrows while restriction sites for *Hae *III are indicated by red arrows.

Sequence analysis revealed that all potential recombinant regions located in the regions of 5.8S and ITS2 (Figure 3, for sequences involved in the recombinant regions, see Figure 2, shown in black background). These potential recombinant regions involved sequences of 9-116 bp, suggesting that recombinant variants may have resulted from site-specific recombination. Biased gene conversion in the hybrid may be drawn by site-specific recombination between the parental ITS sequences. Among these recombinant variants, RV1 and RV2 are mirror complement with exactly the same recombinant region: RV1 had its first part coming from the maternal allele and the second part coming from the paternal allele, while RV2 had the exactly opposite components with the first part coming from the paternal allele, and the second part coming from the maternal allele. This suggested that gene conversion tracts were bidirectional. However, the mirror complement of RV3-RV6 was not found, although no extensive efforts were devoted for the search and they could have also existed.

The Recombinant Variants Were Likely Amplified from Genuine Recombinant Templates: We had to consider the possibility of PCR-generated errors because all the sequenced clones were derived from PCR amplified material. While point mutations introduced by PCR as a result of infidelity of *Taq *polymerase are well documented, and is not the major concern in this situation, template jumping is of concern because it also can interpret the observed recombinant variants. In vitro recombination occurs when incompletely extended PCR segments from one allele serve as primer during subsequent amplification cycles amplified segments [15]. In spite of the technical difficulties in providing absolute proofs because our approaches nonetheless still involved the use of PCR, several considerations made us to believe that the observed recombinant variants were real, and were not derived from PCR artifacts. First, we conducted sequence analysis of the PCR templates for the presence of hairpins or other major secondary structures, and found no major hairpins in the observed recombinant regions except minor secondary structures with RV1, RV2 and RV3. The presumption is that certain partially extended PCR products would have been quite abundant if they had served as primers for subsequent rounds of PCR to allow their sufficient amplification for detection using cloning with limited numbers of sequenced clones. In order to have abundant partially amplified products, secondary structure would have been required. Second, we conducted PCR directly using primers representing the recombinant variants, and PCR products were readily produced. If the recombinant variants had been absent from the template pool, success of such PCR would have required template jumping. To provide a reasonable control, we have conducted PCR amplification using a mixture of DNA isolated from maternal *C. farreri *and paternal *A. irradians *as templates. No PCR products were generated from such mixed templates, suggesting that PCR templates for the recombinant variants were genuinely present in the hybrid scallops. Third, we considered mathematical assessment of product abundance. In this regard, all the recombinant variants but RV5 requires just one recombination while RV5 requires two recombination, and they were all readily detectable from limited sequenced clones. If indeed template jumping was involved, molecules requires more than one recombination should be very rare as it can be picked up several rounds later in subsequent PCR cycles. In addition, the molar ratio of the partially extended products to the PCR primers would be miniscule, and annealing of long, partially extended products would otherwise require more time than the annealing of short PCR primers. This consideration, however, is just theoretical as opposite conclusions were reached by Bradley and Hillis with 29% each of the parental types and 43% of recombinant variants, several of which required more than one recombination (see discussions below).

**Figure 4:**
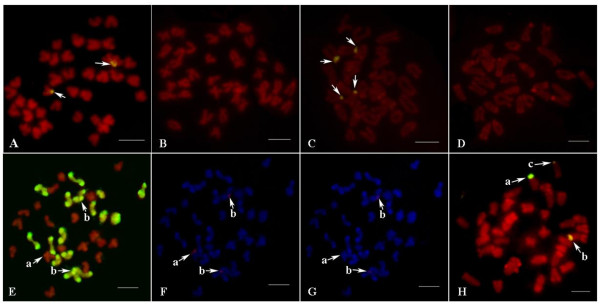
**Chromosomal locations of the parental ITSs in the hybrid**. (A) FISH in *C. farreri *with probe from *C. farreri *(signals are shown by arrows); (B) FISH in *C. farreri *with probe from *A. irradians *(no signal is detected); (C) FISH in *A. irradians *with probe from *A. irradians *(signals are shown by arrows); (D) FISH in *A. irradians *with probe from *C. farreri *(no signal is detected); (E) GISH in the hybrid with genome probe from *A. irradians *(a shows the ITS-bearing chromosome of *C. farreri*; b show the ITS-bearing chromosomes of *A. irradians*; metaphases in E, F and G are same); (F) FISH in the hybrid with probe from *C. farreri *(signals are detected at a and b); (G) FISH in the hybrid with probe from *A. irradians *(no signal is detected at a but weak signals are detected at b); (H) FISH in the hybrid with probe from *C. farreri *(a shows the ITS-bearing chromosome of *C. farreri*; b, c show the ITS-bearing chromosomes of *A. irradians*; signal intensity at b is higher than that at c). Scale bar represents 5 μm.

Cellular Evidence for Maternal Biased Gene Conversion of ITS Alleles: Two competing hypotheses can explain the reduction of the paternal ITS allele in the hybrid: (i) the rDNA-containing chromosomes of *A. irradians *have been gradually lost in the hybrid, since interspecific hybridization is commonly accompanied by chromosome expulsion, or (ii) biased gene conversion which makes the paternal ITS be gradually converted to the maternal ITS, occurred in rDNA family of the hybrid. In order to differentiate the two possibilities, fluorescent in situ hybridization (FISH) and genomic *in situ *hybridization (GISH) were conducted to determine the status of ITS on chromosomes (Figure 4). It has been reported that rDNA family of *C. farreri *is solely located on the telomeric region of the short arm of chromosome 5, while that of *A. irradians *is located on the telomeric region of the short arm of chromosome 4 and chromosome 8 [16]. Because of the significant levels of sequence divergence, (sequence homogeneity less than 80%), the parental ITS sequences can be distinguished by FISH technique under the optimized condition, which allowed us to gain direct cellular evidence for the biased gene conversion.

Chromosomal lose was not involved in the observed reduction of the paternal allele. GISH analysis revealed that the haploid chromosomes from both parents were intact in the hybrid in more than 66% metaphases (see Fig. 4E). In FISH analysis, when probe from *C. farreri *was used for hybridization with the hybrid chromosomes, one signal was found in the ITS-bearing chromosome of *C. farreri *as expected, while two signals were found in the ITS-bearing chromosomes of *A. irradians *(see Fig. 4F), suggesting the presence of *C. farreri *ITS allele sequences on the ITS-bearing chromosomes of *A. irradians*. Moreover, intensities of the two signals that found in the ITS-bearing chromosomes of *A. irradians *were significantly inconsistent in some metaphases (see Fig. 4H). When probe from *A. irradians *was used, only two weak signals were found in the ITS-bearing chromosomes of *A. irradians *as expected, while no signal was found in the ITS-bearing chromosome of *C. farreri *(see Fig. 2G). Taken together, these results provide cellular evidence for the maternal biased gene conversion at the ITS region of the rDNA loci, which confirm the molecular evidence for maternal biased gene conversion.

## Discussion

This work took advantage of the hybrid from two scallop species *C. farreri *and *A. irradians*, whose ITS sequences are readily distinguishable, for the study of concerted sequence homogenization of the rDNA. As reviewed by Eickbush and Eickbush [3], unequal crossover has been regarded as the major mechanism for the evolution of rRNA genes. Here, we provided strong molecular and cellular evidence for maternal biased gene conversion, leading to reduction of the paternal allele toward homogenization with the maternal allele. Although unequal crossover remains a possibility, it does not support to explain the observed sequence homogenization. First, it is difficult to explain why paternal ITS sequences can be totally replaced just through mitotic cell divisions by unequal crossover. Second, about 70% *C. farreri *eggs can be fertilized with *A. irradians *sperm. About 80% fertilized eggs can be developed to trochophore larvae, which is comparable to that observed in the intra-specific cross of *C. farreri*. We used trochophore larvae at the early stage for chromosome spread preparation, and yet, no signal was observed on the maternal rRNA loci using paternal sequence as the probe in the FISH experiment, which suggests unequal crossover did not occur between paternal and maternal rRNA loci-containing chromosomes. Although most theoretical and experimental studies on concerted evolution of rDNA family were conducted in model organisms such as yeast, *Drosophila*, and mice as well as in humans [1], non-traditional model system can provide cleaner evidence for the mechanisms involved in sequence homogenization, as demonstrated here with the scallops. In this paper, maternal biased conversion of ITS sequences was evident during early stages of development of the hybrid scallop. Hybrid larvae at later developmental stages have not been obtained due to high mortality rate, and are therefore not included in the current study.

### Site-specific recombination and double-strand breaks (DSBs)

In yeast, DSBs are considered as the sole mechanism of recombination in meiotic cells and are a major factor in recombination in mitotic cells [9]. In a general model, DSBs are cut on both strands to create large gaps flanked by rather short regions of single-stranded DNA that could invade a homologous template and initiate DNA repair[17]. In this study, potential recombinant regions identified in the hybrid are also conservative in the ITS sequences of other 6 species of *Pectinidae *which represent all now available in GenBank. Recombination in these regions may be site-specific but not sequence-specific since common sequences of these regions are not found. Some sites in these regions may be recognized by some endonucleases, which may be analogous to Spo11p in *Saccharomyces cerevisiae *[18,19], to generate DSBs. In general, homologous recombination in DSB repair involves hundreds of nearly perfectly matched base pairs. However, most recombinant regions identified in this study are very short. It is not unique since homologous recombination in DSB repair can also occur with surprisingly short homologous regions in yeast [9]. Moreover, conversion tracts in RV3-RV6 could be unidirectional because the counterparts of these recombinant variants were not found. The phenomenon that conversion tracts extended only on one side of the DSB was also reported in yeast in meiosis and mitosis [20-22].

Although identification of recombination variants have also been reported in ITS1 sequences of *Darwinula stevensoni *[23], there still exists the possibility that recombinant variants are artifacts generated by PCR amplification [15]. In order to exclude this possibility, PCR product amplified from the mixture of genomic DNA of *C. farreri *and *A. irradians *was cloned and recombination variants were subsequently screened in 300 randomly selected colonies. Only RV1 and RV2 were identified at significantly lower frequency, which suggests that short homologous regions in RV3-RV6 may be not significant to introduce recombination in PCR amplification. Moreover, as an alternative technique, loop-mediated isothermal amplification (LAMP) developed by Notomi et al. [24] was also used in this study (data not shown). Because cycles of denaturation in PCR amplification are not needed in this technique, this technique can significantly avoid the generation of recombinant variants. When specific primers were designed for RV6 (FIP: 5'-ACAGCCGACCCTCAGACA-CATCGATATCTTGAACGCACA

TTGC-3'; BIP: 5'-CCGGCGAGCGGTCTTAAA-CTGGTTTGTTTTTGGTTCGATTG

GA-3'; F3: 5'-TGTGAATTGCAGGACACATTGA-3'; B3: 5'-GCGTCTCTTGTAATTT

GTTCCGTA-3'), RV6 could be amplified from the hybrid and further was confirmed by subsequent sequencing.

Biased gene conversion: Gene conversion can be bidirectional as those reported in *Gossypium *allopolyploid and *Nicotiana *allopolyploid [6,25]. However, gene conversion is biased in the hybrid in this study and similar phenomenon was also reported in other studies [26-28]. This phenomenon may be general in *Pectinidae*, because biased gene conversion was also observed in other hybrids such as *C. farreri *♀ × *Chlamys nobilis *♂, *C. farreri *♀ × *Patinopecten yessoensis *♂, and *P. yessoensis *♀ × *A. irradians *♂ (our unpublished data).

The possible mechanism for biased gene conversion in the hybrid may involve maternal restriction systems. In the initial formation of the hybrid, maternal enzymatic system may treat the chromosomes of *A. irradians *as intruding chromosomes. Specific sites in paternal recombinant regions are thus recognized by maternal endonucleases to produce DSBs. Biased gene conversion can subsequently occur when the directionality of DSB repair is in favor of the maternal DNA sequences. There were evidences from yeast that the directionality of DSB repair is in favor of the donor sequences [29,30].

### The rate of gene conversion and mitosis

Although gene conversion was almost complete in all individuals sampled at the late stage of umbo larvae (about 14 days after fertilization), this process could have started well within 20 hours after fertilization (see Fig. 1). The rate of gene conversion is surprisingly rapid in the hybrid. For rDNA family, although the highest rate calculated from hybrids of *Armeria *was one order of magnitude above that in *Drosophila *reported by Dover [31], one generation is still needed [26]. Liao et al. [32] demonstrated that it is even possible that concerted evolution could be achieved quite rapidly, perhaps within one or a few meioses or mitoses. Because of absence of meiosis, the process of gene conversion in this study thus may occur in mitosis. Rapid gene conversion may be involved with long conversion tracts and/or at high frequency. In mitosis, conversion tracts in yeast could even extend up to 400 kb [9,33]. In general, exchange rates between rDNA arrays residing in the heterochromatin were expected to be rather low. However, it was found that recombination between the rDNA arrays occurs at a much higher rate than the rest regions of the heterochromatin in *Drosophila melanogaster *[34]. It has been suggested in some studies that gathering of rDNA in/at the nucleolus and its exposure to nucleolin play an important role in recombination [35-37].

### Gene conversion between non-homologous chromosomes

It is currently unclear that which of rDNA-containing chromosomes of *A. irradians *is homologous to that of *C. farreri*. Inconsistent numbers of rDNA locus in the two species suggest that at least one of rDNA-containing chromosomes of *A. irradians *is nonhomologous to that of *C. farreri*. Based on the result that two signals were found in the ITS-bearing chromosomes of *A. irradians *in the hybrid when probe from *C. farreri *was used, biased gene conversion had occurred between nonhomologous chromosomes as well as between the homologous chromosomes in the hybrid. Recombination between nonhomologous chromosomes may be more convenient in the hybrid since rDNA loci in the parents are all located on the telomeric region of short arm of chromosomes. Gene conversion among rDNA loci on nonhomologous chromosomes were also reported in humans and apes [38,39]. In the hybrid, intensities of two signals were found significantly inconsistent in some metaphases, which may imply that the rates of gene conversion are different between homologous chromosomes and nonhomologous chromosomes. For example, in Fig. 2H, *a *may be more homologous to *b *than to *c*, because signal intensity in *b *is higher than that in *c*.

The maternally biased gene conversion reported here may have important implications for concerted gene evolution among gene families, as the similar mechanism can be used for sequence homogenization in various multigene families. The appearances of recombinant variants could help explain the origin of hybrid zone novel alleles, a phenomenon long puzzled the scientific community [40]. In spite of their interpretation of recombinant variants as being generated from PCR template jumping [15], the high proportion of recombinant (43%) may argue against their conclusion. In our case here, although the frequency of recombinant variants was relatively low, their evolutionary consequences can be highly significant. If such a mechanism is involved in hybrids, or crossbreeds of more distantly related populations, its impact on the emergence of novel alleles would be tremendously high. The biased gene conversion would also have significance implications for genetic imprinting where only one allele from a specific parental origin is expressed. Therefore, the hybrid scallop offers a unique system for further studies involving all of these issues.

## Conclusions

Taken together, these molecular and cellular evidences support rapid concerted gene evolution via maternally biased gene conversion. As such a process would lead to the expression of only one parental genotype, and have the opportunities to generate recombinant intermediates; this work may also have implications in novel hybrid zone alleles and genetic imprinting, as well as in concerted gene evolution. In the course of evolution, many species may have evolved involving some levels of hybridization, intra- or interspecific, the sex-biased sequence homogenization could have led to a greater role of one sex than the other in some species.

## Methods

### Scallop Materials

Several sex-matured individuals of *C. farreri *and *A. irradians *were obtained from an aquacultural hatchery in Penglai, Shandong Province, China. Artificial hybridization (*C. farreri *♀ × *A. irradians *♂) was carried out in a laboratory. Because *A. irradians *is hermaphroditic, sperm was filtered by a sieve cloth (500 meshes) in order to avoid introducing eggs of *A. irradians*. After fertilization, hybrid larvae were reared at 20°C. Samples of hybrid larvae were taken at the 2-cell stage (about 1 hour after fertilization), the trochophore stage (about 20 hours after fertilization), the early stage of umbo larvae (about 4 days after fertilization), the middle stage of umbo larvae (about 10 days after fertilization) and the late stage of umbo larvae (about 14 days after fertilization). The muscles of parental scallops were preserved at -20°C and the larvae were stored in ethanol at 4°C.

### DNA Extraction

Genomic DNA of parental scallops was extracted from frozen muscle tissues with phenol/chloroform extraction as described by Sambrook et al [41]. Larval DNA extraction was described as follows. Larvae were transferred from ethanol to a cavity slide and left until the ethanol evaporated completely. Then the larvae were rinsed and agitated with sterile pure water. In total, 30 larvae at each stage were individually isolated through micro-operation and then transferred on a mounted needle to a 0.2 mL PCR tube containing 10 μL of STE solution (100 mM NaCl; 10 mM Tris-Cl [pH 8.0]; 1 mM EDTA [pH 8.0]; 0.5 mg/mL proteinase K). The tubes were kept at 56°C for 30 min to break the cells and expose the DNA, and then 95°C for 10 min to inactivate proteinase K.

### PCR Amplification

PCR amplifications were set up in a 20 μL volume composed of 100 ng parental genomic DNA or 10 μL of larval DNA solution, 0.2 μM each primer, 2 mM MgCl_2_, 0.2 mM each dNTP, 1 × PCR reaction buffer, and 1 U *Taq *polymerase (Promega Inc., Shanghai, China). According to the primers designed for *Mytilus *mussels [42], a pair of primers (forward: 5' GTTTCTGTAGGTGAACCTG 3'; reverse: 5' CTCGTCTGATCTGAGGTCGGA 3') were used in this study. These anneal at the 3' end of 18S rRNA gene and the 5' end of 28S rRNA gene, amplifying ITS1, 5.8S gene and ITS2. Thermal cycling used a PTC-100 cycler (MJ Research Inc., USA). All PCR cycles began with an initial denaturation at 94°C for 3 minutes, followed by 30 cycles of 94°C 30 s, 54°C 30 s, and 72°C 1 min, and a final extension at 72°C for 10 min.

### PCR-RFLP Analysis

Two restriction enzymes, *Hae *III and *Mbo *I, were used in the PCR-RFLP analysis. Restriction digestions were performed in 10 μl volumes, containing 3 μl of PCR product, 2 U of restriction enzyme and 1 μl buffer supplied by the manufacturer (NEB Inc., USA). The reaction was incubated at 37°C for 6 h and then stopped by inactivating the restriction enzyme at 80°C for 20 min. Restriction digestion products were analyzed by gel electrophoresis through an agarose gel (1.0%).

### Cloning, Sequencing and Sequence Analysis

PCR product of multiple larvae at the trochophore stage was ligated into pMD18-T (Takara Inc., Dalian, China) and subsequently transformed into *Escherichia coli *DH5α cells. Using PCR-RFLP technique, recombinant variants were screened in 500 randomly selected colonies and corresponding colonies were sequenced with a 3730 automatic sequencer (Applied Biosystems Inc., USA). Alignment of the parental and recombinant variants' sequences was performed using the program ClustalX 1.83[43].

### Chromosome Preparations, FISH and GISH Analysis

Following treatment with colchicine (0.01%) for 2 h at room temperature (RT), trochophore were exposed to 0.075 M KCl solution for 30 min and then fixed three times (15 min each) in fresh ethyl alcohol/glacial acetic acid solution (3:1). After being treated with 50% acetic acid, the fixed larvae were dissociated into a cell suspension, and then dropped onto hot-wet slides and air-dried.

In FISH experiments, probes were labeled by PCR with biotin-16-dUTP. Chromosome spreads were pretreated with 100 μg/ml DNase-free RNase A in 2 × SSC for 1 h at 37°C, and then treated with 0.005% pepsin in 10 mM HCl for 10 min at 37°C. Chromosome preparations were denatured in a mixture containing 70% formamide and 2 × SSC at 72°C for 2 min, dehydrated with a chilled ethanol series (70%, 90%, and 100%; 5 min each), and then air-dried. Slides were then incubated with 20 μl of denatured hybridization mix (5 ng/μl probe, 10% dextran sulphate, 250 ng/μl salmon sperm DNA, 50% deionized formamide in 2 × SSC, 80°C for 5 min and cooled immediately) for 16 h at 37°C in a moist chamber. After hybridization, slides were washed three times (5 min each) in 50% formamide in 2 × SSC at 66°C, three times (5 min each) in 1 × SSC at 66°C, once for 5 min in 2 × SSC at room temperature. Hybridized probes were detected with fluorescein-labeled avidin DCS (Vector Laboratories). Chromosomes were counterstained with 1.5 μg/ml propidium iodide (PI) or 4'-6-Diamidino-2-phenylindole (DAPI) in antifade solution (Vector Laboratories). For multiple hybridizations, slides were washed three times by 2 × SSC to remove PI or DAPI before denaturation in the subsequent hybridization. Slides were observed using a Nikon Eclipse-600 epifluorescence microscope equipped with a CCD camera. The signals were collected using appropriate filter sets and LUCIA software (Laboratory Imaging).

In GISH experiments, genomic DNA from A. irradians was used as the template for labeling a probe. The probe was labeled by nick translation with biotin-16-dUTP. All steps in GISH experiments were the same as described for FISH experiments except that the temperature of post-hybridization wash was set as 42°C.

## Authors' contributions

SW and ZB designed the study. SW and LZ conducted the experiment. SW, JH, ZB and ZL conducted data analysis. ZB supervised the entire study. SW, ZB and ZL wrote the manuscript. All authors read and approved the final manuscript.

## References

[B1] Nei M Rooney AP Concerted and birth-and-death evolution of multigene families Annu Rev Genet 2005 39 121 152 10.1146/annurev.genet.39.073003.112240 16285855 PMC1464479

[B2] Dover G Molecular drive: a cohesive mode of species evolution Nature 1982 299 5879 111 117 10.1038/299111a0 7110332

[B3] Eickbush TH Eickbush DG Finely orchestrated movements: evolution of the ribosomal RNA genes Genetics 2007 175 477 85 10.1534/genetics.107.071399 17322354 PMC1800602

[B4] Gonzalez IL Sylvester JE Human rDNA: evolutionary patterns within the genes and tandem arrays derived from multiple chromosomes Genomics 2001 73 3 255 263 10.1006/geno.2001.6540 11350117

[B5] Hughes KW Petersen RH Apparent recombination or gene conversion in the ribosomal ITS region of a Flammulina (Fungi, Agaricales) hybrid Mol Biol Evol 2001 18 1 94 96 11141197 10.1093/oxfordjournals.molbev.a003724

[B6] Kovarik A Matyasek R Lim KY Skalick K Koukalov B Knapp S Chase M Leitch AR Concerted evolution of 185.826S rDNA repeats in Nicotiana allotetraploids Biological Journal of the Linnean Society 2004 82 615 625 10.1111/j.1095-8312.2004.00345.x

[B7] Parkin EJ Butlin RK Within- and between-individual sequence variation among ITS1 copies in the meadow grasshopper Chorthippus parallelus indicates frequent intrachromosomal gene conversion Mol Biol Evol 2004 21 8 1595 1601 10.1093/molbev/msh163 15155800

[B8] Santoyo G Romero D Gene conversion and concerted evolution in bacterial genomes FEMS Microbiol Rev 2005 29 2 169 183 10.1016/j.femsre.2004.10.004 15808740

[B9] Paques F Haber JE Multiple pathways of recombination induced by double-strand breaks in Saccharomyces cerevisiae Microbiol Mol Biol Rev 1999 63 2 349 404 10357855 10.1128/mmbr.63.2.349-404.1999 PMC98970

[B10] Elder JF Jr Turner BJ Concerted evolution of repetitive DNA sequences in eukaryotes Q Rev Biol 1995 70 3 297 320 10.1086/419073 7568673

[B11] Lopez-Pinon MJ Insua A Mendez J Identification of four scallop species using PCR and restriction analysis of the ribosomal DNA internal transcribed spacer region Mar Biotechnol (NY) 2002 4 5 495 502 10.1007/s10126-002-0030-0 14961243

[B12] Schlotterer C Tautz D Chromosomal homogeneity of Drosophila ribosomal DNA arrays suggests intrachromosomal exchanges drive concerted evolution Curr Biol 1994 4 9 777 783 10.1016/S0960-9822(00)00175-5 7820547

[B13] Kovarik A Pires JC Leitch AR Lim KY Sherwood AM Matyasek R Rocca J Soltis DE Soltis PS Rapid concerted evolution of nuclear ribosomal DNA in two Tragopogon allopolyploids of recent and recurrent origin Genetics 2005 169 2 931 944 10.1534/genetics.104.032839 15654116 PMC1449095

[B14] Wang S Bao Z Zhang L Li N Zhan A Guo W Wang L Hu J A new strategy for species identification of planktonic larvae: PCR-RFLP analysis of the internal transcribed spacer region of ribosomal DNA detected by agarose gel electrophoresis or DHPLC J Plankton Res 2006 28 375 384 10.1093/plankt/fbi122

[B15] Bradley RD Hillis DM Recombinant DNA sequences generated by PCR amplification Mol Biol Evol 1997 14 5 592 593 9159937 10.1093/oxfordjournals.molbev.a025797

[B16] Wang Y Guo X Chromosomal rearrangement in pectinidae revealed by rRNA loci and implications for bivalve evolution Biol Bull 2004 207 3 247 256 10.2307/1543213 15616355

[B17] Szostak JW Orr-Weaver TL Rothstein RJ Stahl FW The double-strand-break repair model for recombination Cell 1983 33 1 25 35 10.1016/0092-8674(83)90331-8 6380756

[B18] Bergerat A de Massy B Gadelle D Varoutas PC Nicolas A Forterre P An atypical topoisomerase II from Archaea with implications for meiotic recombination Nature 1997 386 6623 414 417 10.1038/386414a0 9121560

[B19] Keeney S Giroux CN Kleckner N Meiosis-specific DNA double-strand breaks are catalyzed by Spo11, a member of a widely conserved protein family Cell 1997 88 3 375 384 10.1016/S0092-8674(00)81876-0 9039264

[B20] Nelson HH Sweetser DB Nickoloff JA Effects of terminal nonhomology and homeology on double-strand-break-induced gene conversion tract directionality Mol Cell Biol 1996 16 6 2951 2957 8649406 10.1128/mcb.16.6.2951 PMC231289

[B21] Porter SE White MA Petes TD Genetic evidence that the meiotic recombination hotspot at the HIS4 locus of Saccharomyces cerevisiae does not represent a site for a symmetrically processed double-strand break Genetics 1993 134 1 5 19 8514148 10.1093/genetics/134.1.5 PMC1205443

[B22] Sweetser DB Hough H Whelden JF Arbuckle M Nickoloff JA Fine-resolution mapping of spontaneous and double-strand break-induced gene conversion tracts in Saccharomyces cerevisiae reveals reversible mitotic conversion polarity Mol Cell Biol 1994 14 6 3863 3875 8196629 10.1128/mcb.14.6.3863-3875.1994 PMC358753

[B23] Gandolfi A Bonilauri P Rossi V Menozzi P Intraindividual and intraspecies variability of ITS1 sequences in the ancient asexual Darwinula stevensoni (Crustacea: Ostracoda) Heredity 2001 87 Pt 4 449 455 10.1046/j.1365-2540.2001.00927.x 11737293

[B24] Notomi T Okayama H Masubuchi H Yonekawa T Watanabe K Amino N Hase T Loop-mediated isothermal amplification of DNA Nucleic Acids Res 2000 28 12 E63 10.1093/nar/28.12.e63 10871386 PMC102748

[B25] Wendel JF Schnabel A Seelanan T Bidirectional interlocus concerted evolution following allopolyploid speciation in cotton (Gossypium) Proc Natl Acad Sci USA 1995 92 1 280 284 10.1073/pnas.92.1.280 7816833 PMC42862

[B26] Fuertes Aguilar J Rossello JA Nieto Feliner G Nuclear ribosomal DNA (nrDNA) concerted evolution in natural and artificial hybrids of Armeria (Plumbaginaceae) Mol Ecol 1999 8 8 1341 1346 10.1046/j.1365-294X.1999.00690.x 10447874

[B27] Ganley AR Scott B Concerted evolution in the ribosomal RNA genes of an Epichloe endophyte hybrid: comparison between tandemly arranged rDNA and dispersed 5S rrn genes Fungal Genet Biol 2002 35 1 39 51 10.1006/fgbi.2001.1309 11860264

[B28] Hillis DM Moritz C Porter CA Baker RJ Evidence for biased gene conversion in concerted evolution of ribosomal DNA Science 1991 251 4991 308 310 10.1126/science.1987647 1987647

[B29] Leung W Malkova A Haber JE Gene targeting by linear duplex DNA frequently occurs by assimilation of a single strand that is subject to preferential mismatch correction Proc Natl Acad Sci USA 1997 94 13 6851 6856 10.1073/pnas.94.13.6851 9192655 PMC21248

[B30] Ray BL White CI Haber JE Heteroduplex formation and mismatch repair of the "stuck" mutation during mating-type switching in Saccharomyces cerevisiae Mol Cell Biol 1991 11 10 5372 5380 1922052 10.1128/mcb.11.10.5372-5380.1991 PMC361613

[B31] Dover GA Linkage disequilibrium and molecular drive in the rDNA gene family Genetics 1989 122 1 249 252 2731732 10.1093/genetics/122.1.249 PMC1203689

[B32] Liao D Pavelitz T Kidd JR Kidd KK Weiner AM Concerted evolution of the tandemly repeated genes encoding human U2 snRNA (the RNU2 locus) involves rapid intrachromosomal homogenization and rare interchromosomal gene conversion EMBO J 1997 16 3 588 598 10.1093/emboj/16.3.588 9034341 PMC1169662

[B33] Esposito MS Evidence that spontaneous mitotic recombination occurs at the two-strand stage Proc Natl Acad Sci USA 1978 75 9 4436 4440 10.1073/pnas.75.9.4436 360220 PMC336130

[B34] Williams SM Kennison JA Robbins LG Strobeck C Reciprocal recombination and the evolution of the ribosomal gene family of Drosophila melanogaster Genetics 1989 122 3 617 624 2569433 10.1093/genetics/122.3.617 PMC1203735

[B35] Borggrefe T Wabl M Akhmedov AT Jessberger R A B-cell-specific DNA recombination complex J Biol Chem 1998 273 27 17025 17035 10.1074/jbc.273.27.17025 9642267

[B36] Hanakahi LA Dempsey LA Li MJ Maizels N Nucleolin is one component of the B cell-specific transcription factor and switch region binding protein, LR1 Proc Natl Acad Sci USA 1997 94 8 3605 3610 10.1073/pnas.94.8.3605 9108024 PMC20487

[B37] Thyagarajan B Lundberg R Rafferty M Campbell C Nucleolin promotes homologous DNA pairing in vitro Somat Cell Mol Genet 1998 24 5 263 272 10.1023/B:SCAM.0000007129.98789.1f 10696234

[B38] Arnheim N Krystal M Schmickel R Wilson G Ryder O Zimmer E Molecular evidence for genetic exchanges among ribosomal genes on nonhomologous chromosomes in man and apes Proc Natl Acad Sci USA 1980 77 12 7323 7327 10.1073/pnas.77.12.7323 6261251 PMC350495

[B39] Krystal M D'Eustachio P Ruddle FH Arnheim N Human nucleolus organizers on nonhomologous chromosomes can share the same ribosomal gene variants Proc Natl Acad Sci USA 1981 78 9 5744 5748 10.1073/pnas.78.9.5744 6272316 PMC348849

[B40] Bradley RD Bull JJ Johnson AD Hillis DM Origin of a novel allele in a mammalian hybrid zone Proc Natl Acad Sci USA 1993 90 19 8939 8941 10.1073/pnas.90.19.8939 8415634 PMC47476

[B41] Sambrook J Fritsch E Maniatis T Molecular cloning: a laboratory manual 1989 Cold Springs Harbor Laboratory Press, New York 464 467

[B42] Heath D Rawson P Hilbish T PCR-based nuclear markers identify alien blue mussel (Mytilus spp.) genotypes on the west coast of Canada Can J Fish Aquat Sci 1995 52 2621 2627 10.1139/f95-851

[B43] Thompson JD Gibson TJ Plewniak F Jeanmougin F Higgins DG The CLUSTAL_X windows interface: flexible strategies for multiple sequence alignment aided by quality analysis tools Nucleic Acids Res 1997 25 24 4876 4882 10.1093/nar/25.24.4876 9396791 PMC147148

